# Improved Photostability in Fluorinated 2D Perovskite Single Crystals

**DOI:** 10.3390/nano11020465

**Published:** 2021-02-11

**Authors:** Annalisa Coriolano, Laura Polimeno, Milena De Giorgi, Francesco Todisco, Rosanna Mastria, Vincenzo Ardizzone, Lorenzo Dominici, Dario Ballarini, Aurora Rizzo, Giuseppe Gigli, Daniele Sanvitto, Luisa De Marco

**Affiliations:** 1 CNR NANOTEC Institute of Nanotechnology, Via Monteroni, 73100 Lecce, Italy; annalisacoriolano@hotmail.it (A.C.); polimeno.lauraa@gmail.com (L.P.); milena.degiorgi@nanotec.cnr.it (M.D.G.); francesco.todisco@nanotec.cnr.it (F.T.); rosanna.mastria@nanotec.cnr.it (R.M.); v.ardizzone85@gmail.com (V.A.); lorenzo.dominici@nanotec.cnr.it (L.D.); dario.ballarini@nanotec.cnr.it (D.B.); aurora.rizzo@nanotec.cnr.it (A.R.); giuseppe.gigli@unisalento.it (G.G.); daniele.sanvitto@nanotec.cnr.it (D.S.); 2 Dipartimento di Matematica e Fisica E. De Giorgi, Università Del Salento, Campus Ecotekne, via Monteroni, 73100 Lecce, Italy

**Keywords:** 2D perovskites, single crystals, perovskite stability, photoluminescence

## Abstract

Hybrid organic-inorganic perovskites are very promising semiconductors for many optoelectronic applications, although their extensive use is limited by their poor stability under environmental conditions. In this work, we synthesize two-dimensional perovskite single crystals and investigate their optical and structural evolution under continuous light irradiation. We found that the hydrophobic nature of the fluorinated component, together with the absence of grain boundary defects, lead to improved material stability thanks to the creation of a robust barrier that preserve the crystalline structure, hindering photo-degradation processes usually promoted by oxygen and moisture.

## 1. Introduction

Metal halide hybrid perovskites have received great attention in the last decade for many device applications, such as solar cells due to their high power conversion efficiencies or high-performance light emitters, including light-emitting diodes (LEDs) and lasers [1,2,3,4]. Indeed, hybrid perovskites offer emission color tunability [5], high photoluminescence quantum yield (PLQY) [6] and compatibility with low-cost fabrication processes, like wet chemistry synthesis and large scale deposition techniques. 

Although, the performances of hybrid halide perovskites are comparable to that of the most common inorganic semiconductors, there are some unfavourable aspects that hinder their diffusion on the market, the most important of which is their poor environmental stability. 

Recently, an emerging type of hybrid perovskite, the two-dimensional (2D) perovskite, has demonstrated to help in this respect. 

Two-dimensional perovskites are obtained from the slicing of a three-dimensional (3D) lattice along the (001) plane. The shape and the size of the spacer cations determines this crystallographic cut. The general formula of 2D perovskites is A_2_BX_4_, where A is a long-chain monovalent organic cation, B is a divalent metallic cation (for example Pb), X is a halide anion (Cl, Br, or I). The structure of 2D perovskite is composed by alternating organic (A) and inorganic layers (PbI_6_)^4-^ octahedron), where A acts as an electronically insulating organic spacer that strongly confine excitons in the inorganic part, forming a natural multiple quantum well.

In general, 2D perovskites show greater resistance to moisture and heating stress than their 3D counterpart and offer wider structural diversity since the width of the quantum wells and the distance between neighboring wells can be tuned by properly selecting the organic ligands. This allows to modulate the extent of the confinement with consequent tuning of the optical and electronic properties [7,8]. As a result of dielectric confinement between the insulating organic layers, the excitons in 2D perovskite are stable and show strong photoluminescence up to room temperature [9], unlike the artificially constructed quantum well structures of the classical III−V semiconductors (for example, the GaAs-based heterostructures) where the excitons are observable only at low temperature. This favourable property as well as easiness of fabrication and the simplicity of integration into other systems, such as optical microcavity, make 2D perovskites particularly interesting for the investigation of light-matter coupling with the consequent generation of exciton-polariton quasi-particles [4,10,11], and for the development of new optoelectronic devices based on the control of photonic signals [12]. 

Although there are many published papers on light- and oxygen/moisture-induced degradation of 3D perovskites [13,14,15,16,17] very few studies have been carried out on the stability of 2D perovskites [18,19,20]. In a recent work [18], the behaviour of 2D phenethylammonium lead iodide perovskite (PEAI) single crystals under light irradiation was examined. The authors observe the degradation of the material and the resulting decrease in photoluminescence within a few tens of minutes under laser irradiation at 488 nm. The degradation mechanism they hypothesize consists of an evolution of perovskite to lead iodide (PbI_2_) with loss of organic molecules and reduction of crystals thickness. Their proposed method to increase the perovskite stability consists in the encapsulation with hexagonal boron nitride (hBN). The authors observe that after 30 min of continuous laser irradiation, encapsulated perovskite flakes maintain an emission level equal to 60% of the initial value, while the emission from the non-encapsulated perovskites is completely quenched. The effectiveness of this method has been confirmed by other authors [19]. 

An alternative way to improve the stability of 2D perovskites is the tailoring of their composition. It has been reported [20] that both the type of halogen and the nature of organic moieties could play a role in the durability of these hybrid semiconductors. Some studies carried out on 2D or mixed 3D/2D polycrystalline perovskites demonstrated that the fluorination of organic cations has beneficial effects on the stability and photovoltaic performance of solar cells [21,22], although a complete understanding of this behaviour is still lacking. 

It is, thus, important to expand the knowledge on this topic, in order to remove significant barriers on large-scale applications of halide perovskite in various fields of interest. 

In this work, we synthesized two different 2D perovskite single crystals, (PEA)_2_PbI_4_ and (4F-PEA)_2_PbI_4_, and we studied their light-induced local structural and optical changes, in order to understand the mechanisms involved in the instability of 2D perovskite. We observed that both the nature of the organic ligand and the quality of the crystalline layer affect the robustness of the material, opening up to the possibility to effectively exploit 2D perovskites in optoelectronic devices working at room temperature.

## 2. Results

Phenethylammonium-based 2D perovskites exhibit strong green emission and are extensively investigated for many applications in photonics [11,23,24,25,26]. 

**Figure 1:**
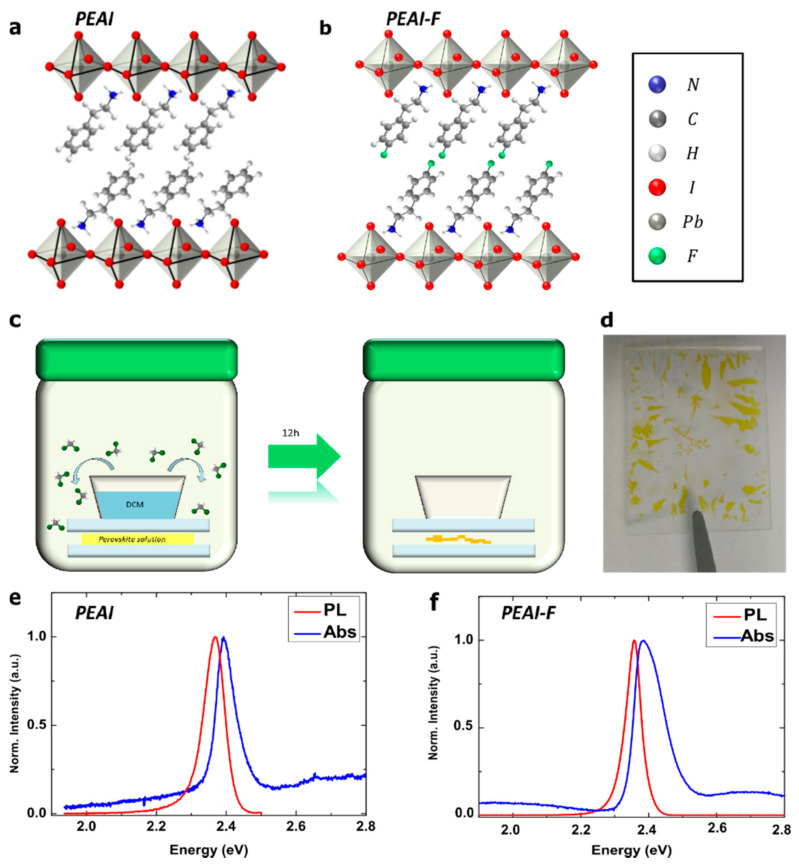
(**a**,**b**) Three-dimensional structure of synthetized PEAI and PEAI-F perovskites. (**c**) Sketch of antisolvent vapor-assisted crystallization method: after 12 h the perovskite solution is fully transformed into perovskite single crystals. (**d**) PEAI single crystals grown on a glass substrate. (**e**) Normalized PL (red line) and Abs (blue line) spectra of PEAI at room temperature. (**f**) Normalized PL (red line) and Abs (blue line) spectra of PEAI-F at room temperature.

In this study, we synthesized phenethylammonium lead iodide perovskite, (PEA)_2_PbI_4_ (henceforth named PEAI), and 4-fluorophenethylammonium lead iodide perovskite, (4F-PEA)_2_PbI_4_ (henceforth named PEAI-F), single crystals. The sketch in Figure 1a,b shows the crystalline structure of PEAI and PEAI-F, which differs only by the presence of a fluorine atom in place of a hydrogen in *para* position on the benzene ring, as determined by X-ray diffraction measurements reported in previously published papers [7,24,27,28]

We investigated the evolution under a continuous wave excitation of single crystals synthesized by the antisolvent vapor-assisted crystallization method [7,8,27]. In this synthesis perovskite precursors (organic cations and PbI_2_ with a molar ratio of 2:1) are dissolved in γ-butyrolactone and dichloromethane is used as antisolvent. As represented in Figure 1e, a few drops of perovskite solution sandwiched between two glass slides are exposed to antisolvent vapours for 12 h at room temperature. As the antisolvent slowly infiltrates the precursor solution, supersaturation is reached and perovskite single crystals begin to form in the gap between the two substrates. At the end of the process, high-quality, millimeter-sized yellow flakes with a thickness of a few microns are obtained (see Figure 1d) which can be mechanically exfoliated to get thin crystals of the desired thickness. The great advantage of this method is that it is simple and straightforward as, with a single-step synthesis at room temperature, higher quality crystals are obtained, which are not affected by heterogeneity and grain boundaries, typical of spin-coated polycrystalline films. 

Figure 1e,f show absorption (Abs) and photoluminescence (PL) spectra taken on thin single-crystal flakes of PEAI and PEAI-F, respectively. The Abs spectra are collected in transmission configuration, exciting the samples with a white Xenon lamp, while a continuous wave 488 nm laser is used for PL measurements (density excitation of 2 W/cm^2^). The Abs peak of PEAI (Figure 1e, blue line) is centered at 2.39 eV while PL (Figure 1e, red line) is at 2.37 eV, with a Stoke shift of 20 meV. PEAI-F shows an Abs peak (Figure 1f, blue line) centered at 2.38 eV and PL peak (Figure 1f, red line) centered at 2.36 eV, with the same Stoke shift of 20 meV. It should be noted that the emission intensity of a PEAI crystal 200 nm thick (200 counts) is higher than that of a PEAI-F crystal having the same thickness (50 counts). It seems that the replacement of a hydrogen atom with a fluorine atom on the benzene ring slightly weakens the emission.

**Figure 2:**
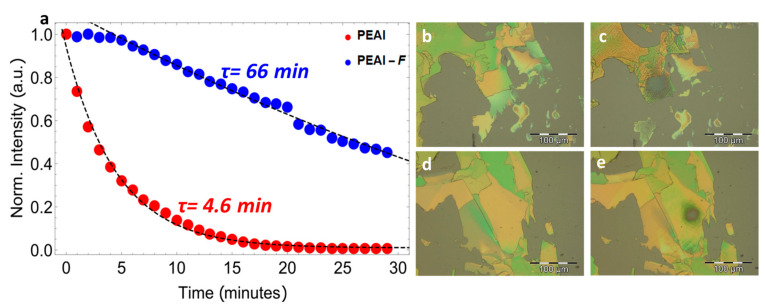
(**a**) Comparison of normalized counts for PEAI (red dots) and PEAI-F (blue dots) as a function of time. Optical image of PEAI single crystal before; (**b**) and after; (**c**) laser irradiation. Optical image of PEAI-F single crystal before; (**d**) and after (**e**) laser irradiation.

To investigate the stability of PEAI and PEAI-F under laser irradiation we have selected two single crystals having the same thickness of 200 nm and we exposed them for 30 min to 488 nm laser irradiation with an excitation density of 2 W/cm^2^. The decrease of the photoluminescence as a function of the irradiation time of the two materials is shown in Figure 2. It is possible to observe the different behaviour of the two perovskites: while the PL intensity of the PEAI (red dots) starts to decrease quickly and shows a decay time (τ) of 4.6 min, PEAI-F (blue dots) exhibits a different trend and shows a decay time of 66 min, demonstrating great robustness and improved stability. The value of the decay time is obtained by fitting the experimental data with a mono-exponential decay function.

It is widely accepted that excitons in 2D perovskites are associated to electron transfer between lead atom and iodide. Therefore, the decrease in PL intensity should be related to detrimental changes that occurs to lead iodide octahedra upon light exposure. It has been reported that perovskites decompose into PbI_2_ and volatile molecules; the degradation mechanism involve light-induced generation of an halide radical (X•), which lead to photodeprotonation of the organic ammonium, with release of volatile components such as amine and HI, leaving PbI_2_ islands in the thin layer [18,20]. 

To explore this issue, we investigated the morphological changes caused by light irradiation in single crystals by optical microscopy. Figure 2b–e display bright field images of PEAI and PEAI-F single crystals before, and after, irradiation, respectively. The surface of freshly exfoliated single crystals is characterized by uniform and smooth flat areas and by the presence of large terraces, typical of layered perovskites. After laser irradiation (488 nm, 2 W/cm^2^) grey defects appear on both materials where the laser beam hit the samples. It seems that the action of the laser is more pervasive on PEAI than on PEAI-F. Indeed, as can be seen from Figure 2c, light-induced morphological changes affect the whole crystal and even portions of crystals in the immediate surroundings. On the contrary, the degradation of PEAI-F (Figure 2e) seems to be circumscribed to the irradiated area. 

To further confirm this observation, scanning electron microscopy (SEM) has been carried out on single crystals exposed at 488 nm laser.

**Figure 3:**
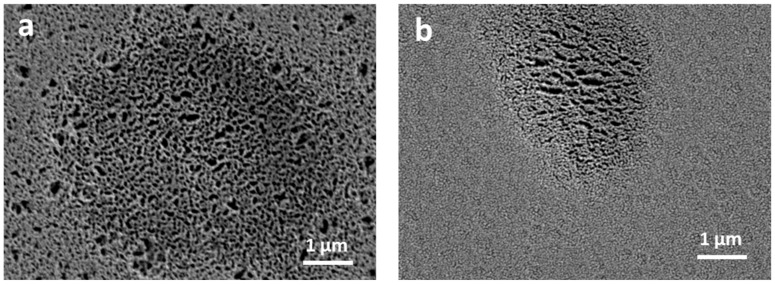
SEM image of PEAI; (**a**) and PEAI-F; (**b**) single crystal after laser irradiation.

SEM images of PEAI and PEAI-F (Figure 3a,b, respectively) show that laser beam induces the formation of defects and holes in both materials, with some differences: on PEAI the area affected by degradation is very large, extending for 6–7 μm, while on PEAI-F the defects appear on an area of 3 μm, comparable with the size of the laser spot. Moreover, pinholes and discontinuities of about 200 nm are clearly visible on the surface of PEAI even outside the laser spot, while on PEAI-F, the surrounding surface seems to be preserved. 

The photodegradation process probably starts at the surface of perovskite crystals where volatile components leave the first layers, breaking the quantum well structure that locally collapse and evolve into PbI_2_ (holes in SEM images). This leads to exposure of new layers to oxygen and moisture and to the generation of defects which promote further damaging of the material. 

The nature of the organic ligand plays a role in this process. The reactions involved in the photodegradation process seem to be favoured by ambient humidity, thus, the introduction of hydrophobic fluorinated compound in PEAI-F may create a more effective barrier that prevents the diffusion of H_2_O through perovskite layers, slowing down and spatially confining the photodegradation. Actually, we observed that in the absence of oxygen and moisture PEAI experiences significantly fewer stability problems than those observed in air (see Figure S1). This suggests that humidity and/or oxygen significantly influence the photodegradation processes in PEAI and have less impact on PEAI-F, supporting the hypothesis that the introduction of the fluorinated ligand may have a beneficial effect in the stability of these materials under environmental conditions. This protection is effective in single crystals, where perovskite thin layers are free of defects and grain boundaries. On the contrary, a polycrystalline film should be more vulnerable, [29] since degradation starts from both the surface and the grain edges of the material and the beneficial effects of fluorinated components could be partially annihilated. 

**Figure 4:**
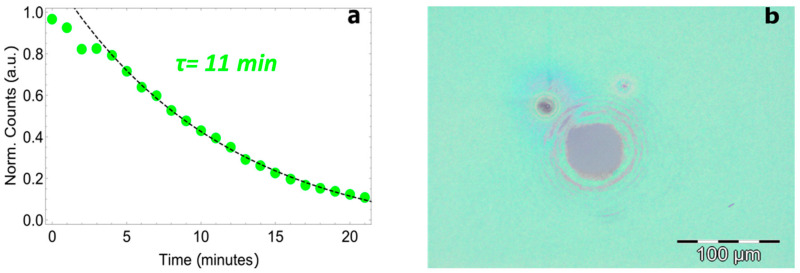
Normalized counts for PEAI-F polycrystalline film as a function of time (**a**). Optical image of PEAI—F polycrystalline film (**b**) after laser irradiation.

To verify this hypothesis, stability measurement was performed on a PEAI–F polycrystalline film having a thickness of 200 nm (Figure 4). We observed faster degradation of the polycrystalline material than the single crystal under the same laser irradiation conditions: spin-coated PEAI-F loses 90% of its initial PL after 20 min. In this case, the curve is fitted by a mono-exponential function with a decay time of 11 min. This demonstrates that a high quality perovskite single crystal is more robust than a polycrystalline film, and confirms the critical role of grain boundary defects in accelerating the H_2_O-triggered photodegradation processes.

## 3. Conclusions

In summary, good robustness has been demonstrated in PEAI-F single crystals under laser irradiation. We attribute the improved stability to both the absence of grain boundaries in single crystals and the hydrophobicity of the fluorinated ligand, which creates an effective barrier that limits the photodegradation processes promoted by ambient moisture. We believe that this strategy could be a way to increase the intrinsic stability of perovskites and can be applied to several applications such as light-emitting diodes, photodetectors and other optoelectronic or nanophotonic devices. 

## 4. Experimental Details

Synthesis of PEAI and PEAI-F single crystals. 

PEAI. Phenethylammonium iodide and lead iodide with a molar ratio of 2:1 were dissolved in gammabutyrolactone, in order to obtain a 1 M solution that was stirred at 70 °C for 1 h, until a clear yellow solution is obtained. 

PEAI-F. 4fluoro-phenethylammonium iodide and lead iodide with a molar ratio of 2:1 were dissolved in gammabutyrolactone, in order to obtain a 0.5 M solution that was stirred at 70 °C for 1 h, until a clear yellow solution is obtained. 

Precursor solutions were prepared in a N_2_—filled glovebox. 2D PEAI and PEAI-F single crystals were synthetized as follow: 3 µL of the perovskite solution was deposited on a glass substrate and then covered by another glass substrate. Dichloromethane (2 mL) was used as antisolvent and placed in a small vial on the top of the substrates which are left undisturbed in a closed Teflon vial for 12 h. Thin and millimeter-sized crystalline flakes of PEAI and PEAI-F are obtained in this way.

Preparation of PEAI-F polycrystalline film.

In a N_2_—filled glovebox 4fluoro-phenethylammonium iodide and lead iodide with a molar ratio of 2:1 were dissolved in *N,N*-Dimethylformamide/Dimethyl sulfoxide 9:1 *v*/*v* in order to obtain a 0.35 M solution. 100 µL of the perovskite solution was deposited on a glass substrate and spin coated for 30 s at 3000 rpm. Dripping with 200 µL of Chlorobenzene was performed at 15 s to the end of the process to increase the quality of the polycrystalline film. In this way a film with a thickness of 200 nm was obtained.

**Figure 5:**
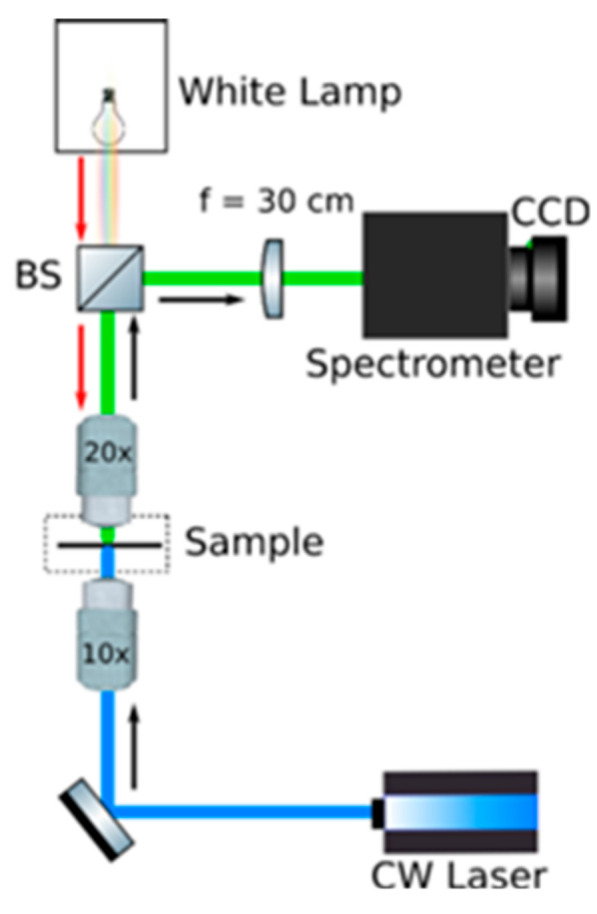
Sketch of the optical setup.

Optical measurements. Figure 5 shows the home-built microscope, used to perform all the optical measurements in air, at room temperature. Photoluminescence is excited through a 10X objective with a continuous wave excitation (488 nm) and the PL signal is collected by a 40X microscope objective, while the absorption spectra are collected exciting the perovskite flakes with a Xenon white lamp, in transmission configuration. The detected signal is focused by a 30 cm lens, into a 300 mm spectrometer (Acton Spectra Pro SP-2300, Princeton Instruments, USA) coupled to a Charge Coupled Device (CCD Pixis eXcelon 400, Princeton Instruments, USA). The spectrometer is equipped with two gratings, 300 g/mm and 1200 g/mm, both of them blazed at 500 nm. In order to evaluate the optical stability, PEAI and PEAI-F single crystals and PEAI-F film are continuously exposed to the laser radiation. The laser power is opportunely reduced by using neutral filters, to obtain a density excitation ≅ 2 W/cm^2^. Optical measurements are carried out in laboratory environment conditions (temperature = 22 °C; relative humidity = 55%).

SEM investigations of 2D perovskite single crystals were performed with a Gemini scanning electron microscope (ZEISS, Germany). The images were acquired at 2 kV accelerating voltages using short exposure times. Samples were grown on Indium Tin Oxide (ITO) conductive glass.

## Data Availability

The data presented in this study are in the paper and/or the Supplemental Information. Additional data related to this paper may be requested from L.D.M. (luisa.demarco@nanotec.cnr.it).

## Supplementary Materials

The following are available online at https://www.mdpi.com/2079-4991/11/2/465/s1, Figure S1: Comparison of normalized counts for PEAI and PEAI-F as a function of time.
