# Supplementary material for: Improved Photostability in Fluorinated 2D Perovskite Single Crystals
Source: Nanomaterials (Basel). 2021 Feb 11;11(2):465. doi: 10.3390/nano11020465 (PMC7918564; doi:10.3390/nano11020465)
Supplement: Supplementary file 1 [file nanomaterials-11-00465-s001.pdf]

nanomaterials

Supporting Information

Improved Photostability in Fluorinated 2D Perovskite Single Crystals

Annalisa Coriolano [1,2], Laura Polimeno [1,2], Milena De Giorgi [1], Francesco Todisco [1], Rosanna Mastria [1], Vincenzo Ardizzone [1], Lorenzo Dominici [1], Dario Ballarini [1], Aurora Rizzo [1], Giuseppe Gigli [1,2], Daniele Sanvitto [1] and Luisa De Marco [1,*]

[1] CNR NANOTEC Institute of Nanotechnology, Via Monteroni, 73100 Lecce, Italy; annalisacoriolano@hotmail.it (A.C.); polimeno.lauraa@gmail.com (L.P.); milena.degiorgi@nanotec.cnr.it (M.D.G.); francesco.todisco@nanotec.cnr.it (F.T.); rosanna.mastria@nanotec.cnr.it (R.M.); v.ardizzone85@gmail.com (V.A.); lorenzo.dominici@nanotec.cnr.it (L.D.); dario.ballarini@nanotec.cnr.it (D.B.); aurora.rizzo@nanotec.cnr.it (A.R.); giuseppe.gigli@unisalento.it (G.G.); daniele.sanvitto@nanotec.cnr.it (D.S.)

[2] Dipartimento di Matematica e Fisica E. De Giorgi, Università Del Salento, Campus Ecotekne, via Monteroni, 73100 Lecce, Italy

* Correspondence: luisa.demarco@nanotec.cnr.it

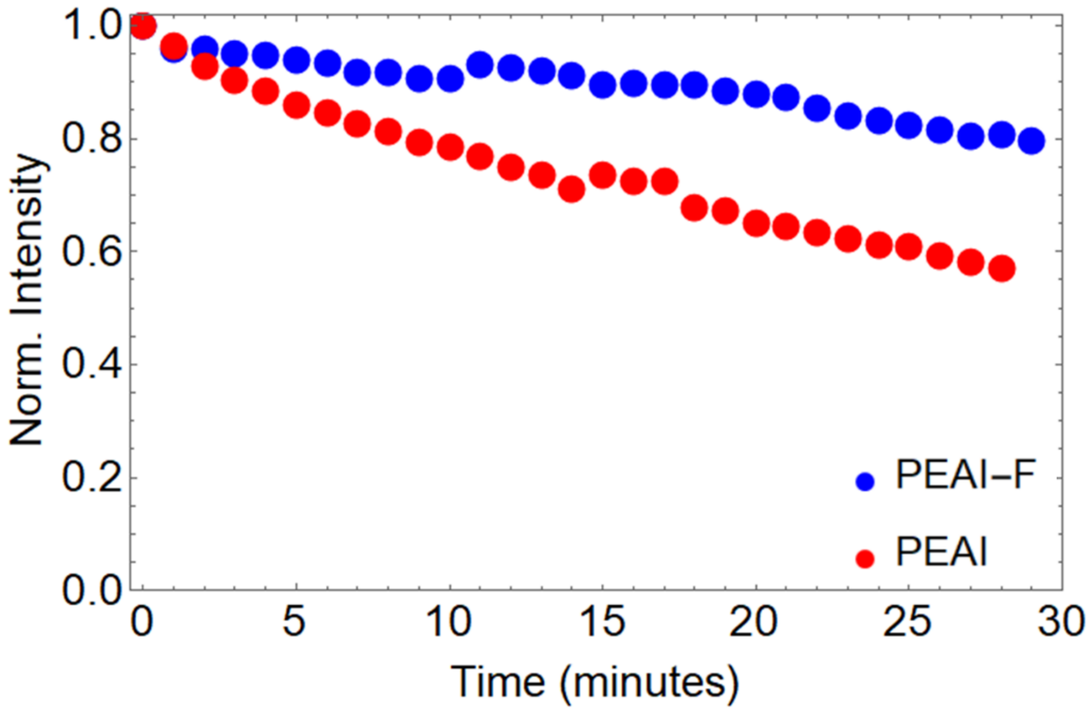

Figure 1. Comparison of normalized counts for PEAI (red dots) and PEAI-F (blue dots) as a function of time. Optical measurements are performed in epiluminescence configuration in a sealed chamber filled with nitrogen. Excitation wavelength 488 nm, density excitation ≅ 4 W/cm^2.
